# Analysis of EGFR, HER2, and TOP2A gene status and chromosomal polysomy in gastric adenocarcinoma from Chinese patients

**DOI:** 10.1186/1471-2407-8-363

**Published:** 2008-12-06

**Authors:** Zhiyong Liang, Xuan Zeng, Jie Gao, Shafei Wu, Peng Wang, Xiaohua Shi, Jing Zhang, Tonghua Liu

**Affiliations:** 1 Department of Pathology, Peking Union Medical College Hospital, Chinese Academy of Medical Science, Beijing 100730, PR China

## Abstract

**Background:**

The EGFR and HER2 genes are located on chromosomes 7 and 17, respectively. They are therapeutic targets in some tumors. The TOP2A gene, which is located near HER2 on chromosome 17, is the target of many chemotherapeutic agents, and co-amplification of HER2 and TOP2A has been described in several tumor types. Herein, we investigated the gene status of EGFR, HER2, and TOP2A in Chinese gastric carcinoma patients. We determined the rate of polysomy for chromosomes 7 and 17, and we attempted to clarify the relationship between EGFR, HER2, and TOP2A gene copy number and increased expression of their encoded proteins. Furthermore, we tried to address the relationship between alterations in EGFR, HER2, and TOP2A and chromosome polysomy.

**Methods:**

One hundred cases of formalin fixed and paraffin embedded tumor tissues from Chinese gastric carcinoma patients were investigated by immunohistochemistry and fluorescence in situ hybridization (FISH) methods.

**Results:**

Forty-two percent of the cases showed EGFR overexpression; 16% showed EGFR FISH positive; 6% showed HER2 overexpression; and 11% showed HER2 gene amplification, including all six HER2 overexpression cases. TOP2A nuclear staining (nuclear index, NI) was determined in all 100 tumors: NI values ranged from 0.5 – 90%. Three percent of the tumors showed TOP2A gene amplification, which were all accompanied by HER2 gene amplification. Nineteen percent of the tumors showed chromosome 7 polysomy, and 16% showed chromosome 17 polysomy. Chromosome 7 polysomy correlated significantly with EGFR FISH-positivity, but was not associated with EGFR overexpression. HER2 overexpression associated significantly with HER2 gene amplification. TOP2A gene amplification was significantly associated with HER2 gene amplification. No relationship was found between alterations in the *EGFR*, *HER2*, and *TOP2A *genes and clinicopathologic variables of gastric carcinoma.

**Conclusion:**

The data from our study suggest that chromosome 7 polysomy may be responsible for increased EGFR gene copy number in gastric carcinomas, and that HER2 gene amplification may be the major reason for HER2 protein overexpression. A combined investigation of the gene status of EGFR, HER2, and TOP2A should facilitate the identification of a target therapeutic regimen for gastric carcinoma patients.

## Background

Gastric cancer is now the second most common cause of cancer death worldwide. Gastric cancer treatment remains a challenge for physicians. Recently, targeted therapy has been applied to gastric carcinoma, which may open new avenues for cancer treatment. Current targeted therapy depends on the evaluation of the status of target genes[1,2].

EGFR and HER2 are members of the epidermal growth factor receptor (EGFR) superfamily with tyrosine kinase activity. EGFR and HER2 are amplified and overexpressed in many human epithelial malignancies, including NSCLC, breast cancer, ovarian cancer, and other forms of cancer; they have both been identified as potential therapeutic targets in several solid tumors, although few reports have focused on gastric carcinoma [3-5]. EGFR and HER2 are located at chromosome bands 7p12 and 17q12-q21, respectively; they encode 185 kDa and 170 kDa plasma membrane glycoproteins, respectively. Previous studies revealed that gene amplification was the main cause of HER2 protein overexpression. However, the reason for EGFR protein overexpression is more complex, it is not known whether EGFR gene copy number correlates with EGFR protein overexpression[3]. Several molecules have been synthesized that inhibit EGFR and HER2 tyrosine kinase domains. These tyrosine kinase inhibitors produced significant responses in advanced NSCLC and breast cancer, and some have been used in the treatment of gastric cancer. Recently, dual inhibition strategies, which target both EGFR and HER2, have shown promising effects against some tumors. Therefore, investigating the gene status of EGFR and HER2 is crucial to determining those patients who would benefit most from target therapies [6-8].

The topoisomerase IIa gene (TOP2A), which is located on chromosome 17q12-q21 near the HER2 oncogene, encodes an enzyme involved in DNA replication. TOP2A is the target enzyme for a specific class of anticancer drugs called anthracyclines. Recent studies have shown that co-amplification of HER2 and TOP2A is associated with sensitivity to anthracycline therapy in several types of cancer. Whether TOP2A gene amplification leads to TOP2A protein overexpression remains controversial [9,10]. A relationship between EGFR and TOP2A has not been reported. Recently, polysomy of chromosome 7, where EGFR resides, was reported to be associated significantly with improved survival after gefitinib treatment in NSCLC patients; based on this finding, chromosome 7 polysomy was considered a predictor for EGFR target therapy[11]. Chromosome 17 aneusomy was common in invasive breast cancer specimens, but correlated primarily with low polysomy 17. Aneusomy for chromosome 17 was not a significant factor for HER2 protein overexpression or for the clinical assessment of HER2 gene status [12]. Previous studies have not shown a relationship between aneusomy for chromosome 17 and TOP2A gene amplification or protein overexpression.

In the current study, EGFR, HER2, and TOP2A gene copy numbers and corresponding levels of protein expression in Chinese gastric carcinomas were determined by FISH and immunohistochemistry(IHC), respectively. Furthermore, polysomy for chromosome 7, where EGFR resides, and for chromosome 17, where HER2 and TOP2A reside, were determined. These experiments allowed the analysis of the relationship between EGFR, HER2, and TOP2A gene copy number and corresponding protein overexpression. The correlation between EGFR, HER2, and TOP2A in gastric carcinomas was determined. Finally, the relationship between chromosome 7 polysomy and EGFR amplification/overexpression, and the relationship between chromosome 17 polysomy and HER2/TOP2A amplification/overexpression were also evaluated.

## Methods

### Tumor tissue collection and human subjects approval

The specimens were selected from archive paraffin embedded blocks in Peking Union Medical College Hospital by two pathologists, 793 Chinese patients with gastric adenocarcinoma who underwent surgery at the Department of Surgery, Peking Union Medical College Hospital were eligible to be selected for the period of 2000–2005. Only those patients whose clinical data (include diagnosis, age, sex, address, disease history, etc) were intact and the blocks were enough to be cut into 20 slides were selected, and patients from different areas of China were included. One hundred Chinese patients with gastric adenocarcinoma were finally selected in this study. As the sample size is small and specimens with small tumor size (not enough to make 20 slides) were excluded, the significance of this study may be limited. Because no previous data from Chinese patients has been published before, we present our data as a reference. If the sample size would have been larger, the results would be more representative, which is a limitation of our study.

The patient group was composed of 82 males and 18 females, with an average age of 60 years. Cancer staging was classified according to the TNM cancer staging system of the American Joint Committee of Cancer[13]: stage IA, 12 cases; stage IB, 7 cases; stage II, 16 cases; stage IIIA, 31 cases; stage IIIB, 11 cases; and stage IV, 23 cases. The World Health Organization Classification of Tumors was used for histologic classification and grading[14]. The institutional review board at the Peking Union Medical College Hospital approved this study, and informed consent was obtained from all patients.

### Immunohistochemistry

All gastric tumor specimens were fixed in 10% buffered formalin and embedded in paraffin according to standard procedures. Serial sections (4 μm thickness) placed on positively charged slides (MENZEL-GLASSER, GERMAN) were used for hematoxylin and eosin staining, immunohistochemistry, and FISH detection of EGFR, HER2, and TOP2A.

Immunohistochemical detection of HER2, EGFR, and TOP2A was determined in all 100 primary gastric cancer tumors. Immunohistochemistry for EGFR and HER2 was performed using the EGFR pharmDx kit (DakoCytomation, Denmark), and the HercepTest kit (DakoCytomation, Denmark), respectively, according to the manufacturer's instructions. A monoclonal antibody against TOP2A (Genetech, Shanghai, China) was used for TOP2A immunohistochemical detection, and TOP2 antigen retrieval was performed by autoclaving in 10 mmol/L citrate buffer (pH 6.0) for 10 minutes at 121.8°C. Antibody binding was visualized by the EnVison detection kit (DakoCytomation, Denmark).

Immunohistochemical staining for EGFR and HER2 was evaluated following the criteria recommended by the manufacturer: 0, no discernible staining or background type staining; 1+, equivocal discontinuous membrane staining; 2+, unequivocal membrane staining with moderate intensity; and 3+, strong and complete plasma membrane staining. More than 30% of the cells were required to meet the criteria for HER2 analysis according to recent published guidelines[15], and more than 10% of the cells were required to meet the criteria for EGFR analysis. Scores of 2+ and 3+ staining levels were considered to be EGFR overexpression, Scores of 3+ staining levels were considered to be HER2 overexpression. Evaluation of TOP2A-positive cells was determined by assessing the proportion of positive tumor cell nuclei within a neoplasm; the percentage of positively stained nuclei was calculated by examining 200 cancer cells and was expressed as the nuclear index (NI).

### FISH analysis

#### EGFR FISH analysis

EGFR FISH analysis was carried out using the LSI EGFR SpectrumOrange/CEP 7 SpectrumGreen probe (Vysis, Abbott Laboratories) according to the manufacturer's protocol. Sections were incubated at 56°C overnight, deparaffinized by washing in CitriSolv (Fisher Scientific, Pittsburgh, PA), and dehydrated in 100% ethanol. After incubation in 2× saline sodium citrate buffer (2× SSC, pH 7.0) at 75°C for 15 – 25 minutes, sections were digested with proteinase K (0.25 mg/mL in 2× SSC; pH 7.0) at 37°C for 15 – 25 minutes, rinsed in 2× SSC at room temperature for 5 minutes, and dehydrated in a series of increasing concentrations of ethanol (70%, 85%, and 100%). The EGFR/CEP 7 probe set was applied to the selected area based on the presence of tumor foci on each slide, and the hybridization area was covered with a glass coverslip and sealed with nail polish. The slides were incubated at 80°C for 8 – 10 minutes for co-denaturation of the chromosomal and probe DNA and were then hybridized at 37°C for 20 – 24 hours. Post-hybridization washes were performed in 1.5 M urea and 0.1× SSC (pH 7.0 – 7.5) at 45°C for 30 minutes and in 2× SSC for 2 minutes at room temperature. After the samples were dehydrated in ethanol as above, 4',6'-diamidino-2-phenylindole (DAPI) in phosphate-buffered saline and glycerol (Vysis) was applied for chromatin counterstaining. FISH analyses were performed independently by two authors who were blinded to the clinical characteristics of the patients and to all other molecular variables. For EGFR FISH analyses, 60 nuclei were scored for signals from both DNA probes using an Olympus BX51TRF microscope (Olympus, Japan) equipped with a triple-pass filter (DAPI/Green/Orange; Vysis) at a final magnification of 1000×.

Chromosome 7 polysomy and monosomy were defined as ≥ three signals and one signal, respectively, in more than 20% of the tumor cells. EGFR gene status was classified into six categories according to the frequency of tumor cells with specific copy numbers of the EGFR gene and the chromosome 7 centromere as described elsewhere [16-18]: disomy (≤ 2 copies in > 90% of cells), low trisomy (≤ 2 copies in ≥ 40% of cells, 3 copies in 10 – 40% of cells, and ≥ 4 copies in < 10% of cells), high trisomy (≤ 2 copies in ≥ 40% of cells, 3 copies in ≥ 40% of cells, and ≥ 4 copies in < 10% of cells), low polysomy (≥ 4 copies in 10 – 40% of cells), high polysomy (≥ 4 copies in ≥ 40% of cells), and gene amplification (presence of tight EGFR gene clusters and a ratio of the EGFR gene to chromosome 7 of ≥ 2, or ≥ 15 copies of EGFR per cell in ≥ 10% of cells). Based on the EGFR gene status, patients were further classified into two groups: 1) EGFR FISH-negative or low gene copy (disomy, low trisomy, high trisomy, and low polysomy), and 2) EGFR FISH-positive or high gene copy (high polysomy and gene amplification). For each FISH preparation, known positive and negative cells were used as controls.

#### TOP2A and HER2 FISH analysis

Three-color FISH to assess the copy number of TOP2A and HER2 was performed using a probe set consisting of SpectrumOrange-labeled TOP2A, SpectrumGreen-labeled HER2, and SpectrumAqua-labeled centromere 17 (Vysis); the CEP 17 probe was used as a chromosomal copy number control. Absolute and relative numbers (relative to chromosome 17 copy number) of the individual genes were scored in at least 40 cancer nuclei per tumor. FISH labeling was performed according to the Vysis protocol and as described previously. Briefly, hybridization buffer, DNA probe, and purified water were combined, centrifuged, and heated to 73°C for 5 minutes in a water bath. Slides were immersed in a denaturing bath (70% formamide/2× SSC) for 5 minutes at 73°C, followed by dehydration in increasing ethanol concentrations, and then dried. The probe mix was applied to each slide, and slides were placed in a 42°C incubator for 30 minutes. Slides were subsequently washed in 0.4× SSC/0.3% NP-40 for 2 minutes, air-dried in darkness, counterstained with DAPI, and a coverslip was applied. All levels for HER2 and TOP2A are reported as a gene/CEP 17 ratio to normalize to the total chromosome number within each cell. Amplification was defined as a gene/CEP 17 ratio of 2.2[15]. Disomy for chromosome 17 was defined as an average of 1.75 to 2.25 CEP17 signals per cell, whereas cases with < 1.75 (hypodisomy) or > 2.25 (polysomy) CEP 17 signals per cell were defined as having aneusomy for chromosome 17[12,19]. For each FISH preparation, known positive and negative cells were used as controls.

### Statistical analysis

Statistical analyses were performed using Pearson's Chi-square test or Fisher's exact test to determine significant clinicopathological differences between EGFR expression in both positive and negative tumors, between EGFR FISH-positive and FISH-negative tumors, between tumors with and without TOP2A overexpression/amplification, and between HER2 expression/amplification in positive and negative tumors; to determine the association between EGFR overexpression and EGFR gene status, between HER2 overexpression and HER2 gene status, and between TOP2A NI and TOP2A gene status; and to investigate the relationship between gene status and chromosomal polysomy. Odds ratio and 95% confidence intervals (CIs) were calculated. Nineteen comparisons were performed, after a Bonferroni adjustment was made to adjust for multiple comparisons, and the difference at P < 0.0026(0.05/19) was considered significant.

## Results

### Correlation with clinicopathologic variables

No significant relationship was found between clinicopathologic variables (sex, age, tumor differentiation, stage, or TNM classification) and EGFR overexpression, EGFR high polysomy/amplification, HER2 overexpression, HER2 amplification, TOP2A overexpression, TOP2A amplification, chromosome 7 polysomy, or chromosome 17 polysomy.

### EGFR status in gastric carcinoma

**Figure 1:**
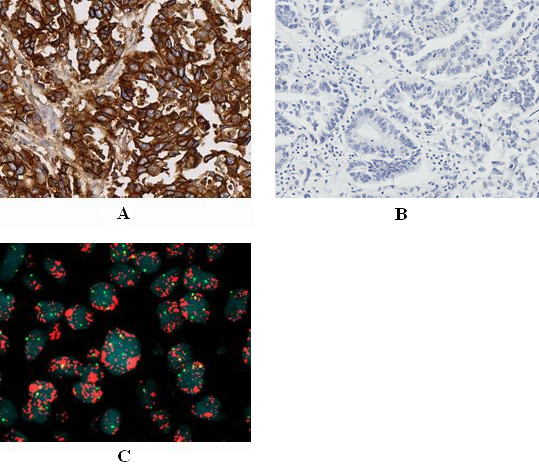
A: Case 10, immunohistochemistry showed EGFR positive(3+) in gastric adenocarcinoma, B: Immunohistochemistry showed EGFR negative(0) control in gastric adenocarcinoma, C: Case 10, FISH result showed EGFR amplification (red signal in cluster) in gastric adenocarcinoma.

Eleven percent of the cases showed EGFR expression levels corresponding to 3+, 31% showed EGFR levels of 2+, and 5% showed EGFR levels of +; the remaining 53% of cases showed level 0 staining (n = 100 gastric tumor samples). Three percent of the cases showed EGFR amplification, 13% showed high polysomy, 28% showed low polysomy, 24% showed low trisomy, 32% showed disomy, and none of the cases showed high trisomy. EGFR overexpression was not associated with EGFR FISH positivity (high polysomy and amplification) (p > 0.0026) (Table 1, Figure 1).

**Table 1:** Relationship between EGFR copy number and EGFR protein expression

EGFR IHC	EGFR FISH						Total
	
	disomy	Low trisomy	High trisomy	Low polysomy	High polysomy	Amplification	
Negative	25	11	0	15	7	0	58
Positive	7	13	0	13	6	3	42
Total	32	24	0	28	13	3	100
P value	>0.0026*						
Odds ratio	1.987						

*: the association between EGFR protein expression and EGFR FISH positive (high polysomy and amplification) and negative (disomy, low trisomy, high trisomy and low polysomy) (P > 0.0026)

EGFR IHC Positive:IHC staining level of 2+and 3+, EGFR IHC Negative:IHC staining level of 0 and 1+.

### EGFR status and chromosome 7 polysomy

Nineteen percent of the cases showed chromosome 7 polysomy; 10 out of the 19 cases with chromosome 7 polysomy showed EGFR high polysomy, and the remaining nine cases showed EGFR low polysomy. Nine of the 19 cases showed EGFR overexpression. Chromosome 7 polysomy showed a significant association with EGFR FISH-positivity after Bonferroni correction (p < 0.0001). Chromosome 7 polysomy showed no association with EGFR overexpression (p > 0.0026) (Table 2).

**Table 2:** Relationship between chromosome 7 polysomy and EGFR copy number/EGFR protein expression

Chromosome 7 copy number	EGFR FISH		EGFR IHC	
	
	Negative	Positive	Negative	Positive
Non-polysomy	74	7	48	33
Polysomy	10	9	10	9
Total	84	16	58	42
P value	<0.0001*		>0.0026^#^	
Odds ratio	8.2286		1.3091	

*: association between chromosome 7 polysomy and EGFR copy number(p < 0.0001), significant after Bonferroni correction;

#: association between chromosome 7 polysomy and EGFR protein expression (p > 0.0026).

EGFR FISH Positive: EGFR high polysomy and amplification. EGFR FISH Negative:EGFR disomy, low trisomy, high trisomy, and low polysomy

### HER2 status in gastric carcinoma

**Figure 2:**
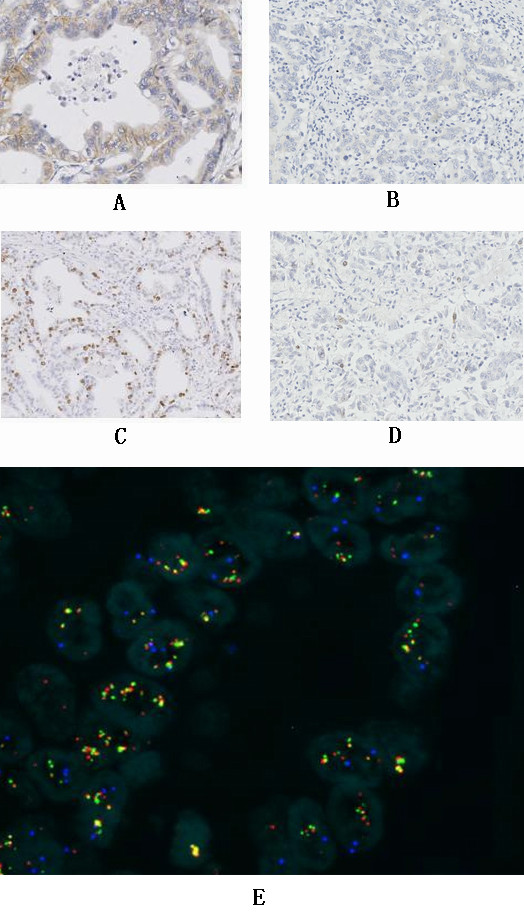
A: Case 36, immunohistochemistry showed HER2 2+ in gastric adenocarcinoma, B: Immunohistochemistry showed HER2 negative control in gastric adenocarcinoma, C: Case 36, Immunohistochemistry showed TOP2A NI (nuclear index) 40% in gastric adenocarcinoma, D: Immunohistochemistry showed TOP2A NI (nuclear index) less than 1% (under expression) in gastric adenocarcinoma, E: Case 36, FISH result showed HER2 (green signal) and TOP2A (red signal) coamplification in gastric adenocarcinoma.

Six percent of the cases showed HER2 3+ levels, and 7% showed HER2 2+ levels. Sixteen percent of the cases showed HER2 1+ levels, and the remaining 71% of the cases showed a 0 level of HER2 staining. Eleven percent of the cases showed HER2 gene amplification; the HER2 amplification cases included all six of the HER2 3+ cases, three of the HER2 2+ cases, and two of the HER2 0 cases by immunohistochemistry. HER2 overexpression (3+) showed a significant association with HER2 amplification after Bonferroni correction (p < 0.0001) (Table 3, Figure 2).

**Table 3:** Relationship between *HER2 *amplification and HER2 protein expression.

HER2 IHC	HER2 FISH		Total
	
	Negative	Positive	
Negative	89	5	94
Positive	0	6	6
Total number	89	11	100
P value	<0.0001*		
Odds ratio	211.546		

Positive: HER2 amplification, Negative: No HER2 amplification.

*: significant after Bonferroni correction.

### HER2 status and chromosome 17 aneusomy

Sixteen percent of the cases showed chromosome 17 polysomy, 8 percent of the cases showed chromosome 17 hypodisomy, the other 76 percent of the cases showed disomy. One of the 16 cases with chromosome 17 polysomy showed HER2 amplification and HER2 IHC levels of 3+. There was no association between chromosome 17 polysomy and HER2 amplification (p > 0.0026), and there was no significant association between chromosome 17 polysomy and HER2 overexpression (p > 0.0026) (Table 4).

**Table 4:** Relationship between chromosome 17 copy number and HER2 amplification/HER2 protein expression

Chromosome 17 Copy number	HER2 FISH		HER2 IHC	
	
	Negative	Positive	Negative	Positive
Non-polysomy	74	10	79	5
Polysomy	15	1	15	1
Total	89	11	94	6
P value	>0.0026*		>0.0026^#^	
Odds ratio	0.4933		1.0533	

*: Association between chromosome 17 polysomy and HER2 amplification.

#: Association between chromosome 17 polysomy and HER2 protein expression.

HER2 FISH Positive: HER2 amplification, HER2 FISH Negative: No HER2 amplification,

HER2 IHC Positive: IHC staining level of 3+, HER2 Negative: IHC staining level of 0,1+,2+

### TOP2A status in gastric carcinoma

**Figure 3:**
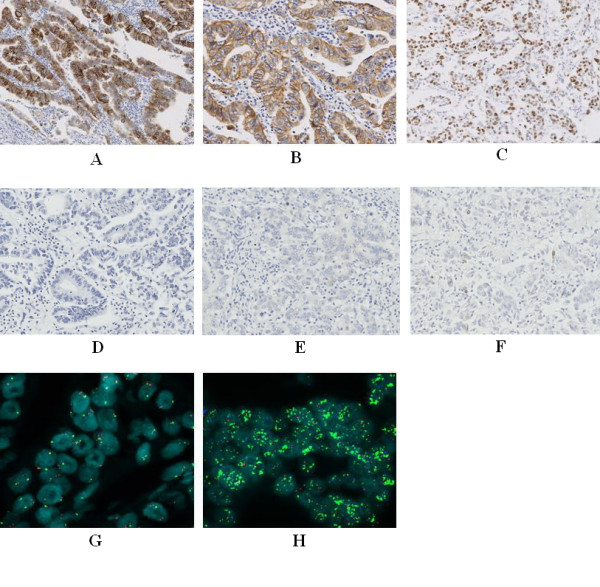
**A: Case 6, immunohistochemistry showed EGFR 3+ in gastric adenocarcinoma, B: Case 6, immunohistochemistry showed HER2 3+ in gastric adenocarcinoma, C: Case 6, immunohistochemistry showed TOP2A expression NI 80% in gastric adenocarcinoma.** D: Immunohistochemistry showed EGFR negative(0) control in gastric adenocarcinoma, E: Immunohistochemistry showed HER2 negative control in gastric adenocarcinoma, F: Immunohistochemistry showed TOP2A NI (nuclear index) less than 1% (under expression) in gastric adenocarcinoma G:case 6, FISH result showed EGFR high polysomy (red signal) in gastric adenocarcinoma, H:case 6, FISH result showed HER2 amplification (green signal in cluster), TOP2A FISH negative (red signal) in gastric adenocarcinoma.

**Figure 4:**
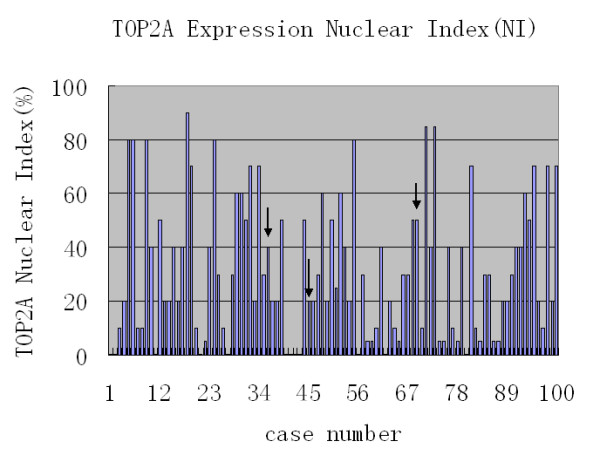
TOP2A expression nuclear index in 100 cases of gastric carcinoma, NI value is from 0.5 to 90%, three cases showed TOP2A amplification (arrows) with TOPNI values of 20%, 40%, and 50%.

TOP2A protein expression was detected in all 100 tumors: NI values ranged from 0.5 – 90%, and three cases showed TOP2A amplification with NI values of 20%, 40%, and 50%, respectively (Figure 3, 4). No TOP2A deletions were detected in any of the 100 tumor specimens. None of the 16 chromosome 17 polysomy cases showed TOP2A amplification.

To determine whether the TOP2A expression evident in gastric adenocarcinomas was a result of gene amplification or chromosome 17 polysomy, we established three cut-off points for TOP2A NI values at 5%, 10%, and 25%, and tried to identify the statistical difference in the prescence of TOP2A amplification or chromosome 17 polysomy with a cut-off less than 5%(or10%,25%) vs 5% (or10%,25%)or more. No correlation was found between TOP2A amplification and TOP2A NI values (p > 0.0026) (Table 5). No association was found between choromosome 17 polysomy and TOP2A NI values (p > 0.0026). No significant association was found between chromosome 17 polysomy and TOP2A amplification (p > 0.0026) (Table 6) (Figure 2).

**Table 5:** Relationship between TOP2A gene amplification and TOP2A protein expression

TOP2A NI	TOP2A FISH		Total	P value	Odds ratio
				
	Negative	Positive			
<5%	12	0	12	>0.0026	1.0234
≥5%	85	3	88		

<10%	22	0	22	>0.0026	2.0861
≥10%	75	3	78		

<25%	48	1	49	>0.0026	1.9592
≥25%	49	2	51		

Positive: TOP2A amplification, Negative: No TOP2A amplification.

**Table 6:** Relationship between chromosome 17 copy number and TOP2A complification/TOP2A protein expression

Chromosome 17	TOP2A FISH		TOP2A NI					
	
	Negative	Positive	<5%	≥5%	<10%	≥10%	<25%	≥25%
Non-polysomy	81	3	11	73	20	64	52	32

Polysomy	16	0	1	15	2	14	2	14

Total	97	3	12	88	22	78	49	51

P value	>0.0026*		>0.0026^#^		>0.0026^#^		>0.0026^#^	

Odds ratio	0.7056		2.2603		2.1875		4.3076	

*: Association between chromosome 17 polysomy and TOP2A amplification.

#: Association between chromosome 17 polysomy and TOP2A NI value at three different cut-off points (<5%vs ≥ 5%, <10% vs ≥ 10%, <25%vs ≥ 25%)

Positive: TOP2A amplification, Negative: No TOP2A amplification.

### Correlation between EGFR status and HER2 status

Among the 13 tumors with HER2 overexpression, seven (53.8%) showed EGFR overexpression. There was no significant relationship between HER2 expression and EGFR overexpression (p > 0.0026). Among the 11 tumors with HER2 amplification, three showed EGFR high polysomy, none showed EGFR gene amplification, and there was no significant relationship between HER2 gene amplification and EGFR high polysomy and/or EGFR gene amplification (p > 0.0026) (Figure 3).

### Correlation between EGFR status and TOP2A status

There was no association between TOP2A NI levels and EGFR overexpression (p > 0.0026). Among the three tumors with TOP2A gene amplification, one showed EGFR high polysomy, none showed EGFR gene amplification, and there was no association between TOP2A gene amplification and EGFR high polysomy and/or EGFR gene amplification (p > 0.0026) (Figure 3).

### Correlation between HER2 status and TOP2A status

There was no significant relationship between TOP2A NI levels and HER2 overexpression (p > 0.0026). Among the three tumors with TOP2A gene amplification, two showed HER2 gene amplification, demonstrating a significant correlation between TOP2A amplification and HER2 amplification (p < 0.0001) (Figure 2, 3), still significant after Bonferroni correction.

## Discussion

In this study, we demonstrated that EGFR, HER2, and TOP2A were overexpressed and amplified in gastric carcinomas from Chinese patients; similar results were observed in Western countries and in other Asian countries [19-22]. Therefore, it is reasonable to focus on EGFR, HER2, and TOP2A overexpression and/or gene amplification when developing therapeutic strategies to target gastric carcinomas. Furthermore, it will be important to evaluate the status of the EGFR, HER2, and TOP2A genes in patients prior to treatment.

Previous studies to determine whether high EGFR copy number was associated with EGFR overexpression have been controversial [23-26]. Our results showed that EGFR FISH-positive status did not correlate with EGFR overexpression, further studies will be required to confirm their relationship.

Abnormalities in gene copy number or structure due to chromosomal aneusomy or DNA aneuploidy may lead to altered gene dosage and can directly affect the transcriptional programs for some tumor-associated genes, however an increase in DNA copy number alone does not always lead to transcriptional up-regulation[9]. Here, we provide the first report of chromosome 7 aneusomy in gastric carcinomas. Our results revealed that chromosome 7 polysomy was a frequent event in gastric carcinoma. We found that chromosome 7 polysomy associated strongly with high EGFR copy number. In contrast to a previous report in lung cancer[11], we did not observe an association between chromosome 7 polysomy and EGFR overexpression. The mechanism underlying EGFR overexpression remains to be clarified.

Chromosome 17 harbors a number of important oncogenes and tumor suppressor genes, including HER2, TOP2A, DARPP32, p53, and BRCA1[21]. In the present study, all six HER2 3+ cases showed HER2 gene amplification, and three HER2 2+ cases showed HER2 gene amplification. Therefore, HER2 gene amplification correlated significantly with HER2 overexpression; this finding is consistent with previous reports [19-21]. Chromosome 17 polysomy did not correlate with HER2 amplification or with HER2 overexpression. Rather, our results suggest that increased HER2 gene dosage resulting from gene amplification is the most important determinant of HER2 overexpression, whereas any influence resulting from polysomy for chromosome 17 alone is unlikely to play a significant role in HER2 gene overexpression at the transcriptional level; a similar finding was described previously in breast cancer [12].

The EGFR superfamily has four members: EGFR/HER1, HER2/c-erbB-2/NEU, HER3, and HER4. The EGFR superfamily members are activated by dimerization of identical receptors (homodimerization) or by dimerization of different family members (heterodimerization). No specific ligand has been identified for HER2, which always forms heterodimers with EGFR, HER3, or HER4; HER2-associated heterodimers showed an increased capacity for translating mitogenic signals relative to homodimers, and the heterodimers showed a synergistic effect on cellular transformation. It has been suggested that drugs designed to target either EGFR or HER2 would be effective at least in HER2 amplification patients; however, EGFR and HER2 do not always show high gene copy number or high protein levels at the same time. Therefore, dual inhibition therapy designed to target EGFR and HER2 together may prove to be an ideal regimen for EGFR- or HER2-positive cancer patients. Very recently, small tyrosine kinase inhibitors that simultaneously block EGFR and HER2 have been identified [7-9]. In the present study, we did not identify a correlation between EGFR and HER2 protein expression and gene copy number in gastric carcinomas: only 7 cases showed co-expression of EGFR and HER2, and 3 cases were simultaneously FISH-positive for EGFR and HER2. Dual inhibitor of EGFR and HER2 may be benefit to patients with gastric carcinoma.

TOP2A is among the genes reported to be co-amplified in the HER2 amplicon at 17q12-21, and alterations in TOP2A gene copy number are well studied because it is a molecular target of many anti-cancer drugs termed topo II inhibitors, including anthracyclines, epipodophyllotoxins, actinomycxin, and mitoxantrone[27,28]. Although TOP2A inhibitor therapy is dependent on TOP2A expression levels, whether TOP2A gene amplification is associated with TOP2A overexpression remains controversial[9]. Varis et al [21] reported that TOP2A gene amplification and increased protein expression were concordant, suggesting that gene amplification was largely responsible for TOP2A overexpression. Our study showed that TOP2A gene amplification detected by FISH did not correlate with the TOP2A immunohistochemistry score. This discrepancy may be due to cell cycle-dependent expression of TOP2A, which peaks at the G2/M phase of the cell cycle and declines to a minimum at the end of mitosis; the immunohistochemical analysis only determined the number of proliferating cells, not the differences in the TOP2A expression levels, so the IHC score could not be used as an indicator for TOP2A inhibitor chemotherapy. Previously published reports suggest that TOP2A amplification and deletion account for relative chemosensitivity and chemoresistance, respectively, to TOP2A inhibitor therapy depending on the specific genetic defect at the TOP2A locus in breast carcinoma [29]. We did not detect TOP2A deletions in the present study.

In our study, TOP2A amplification was not a frequent event in gastric carcinoma; this may be the reason why anthracycline therapies have not been effective in targeting gastric carcinomas. Previous studies showed that TOP2A and HER2 were co-amplified in approximately 30–90% of breast carcinomas; the underlying molecular mechanism for co-amplification is not known [9]. Many reports have shown that HER2 and TOP2A co-amplification predicted a good response to TOP2A inhibitor chemotherapy in breast carcinoma, and that a combined therapy with HER2 inhibitors and TOP2A inhibitors would be a good choice for the treatment of these cancer patients. Our studies showed that TOP2A amplification was associated strongly with HER2 amplification in gastric carcinoma: among the ten HER2 amplification cases, two (20%) showed TOP2A amplification, whereas one TOP2A amplification case showed no HER2 amplification showing that TOP2A could be amplified independent of HER2 in gastric carcinoma. Collectively, the results of this study are consistent with previous reports of other tumor types [30-32].

We were unable to determine a relationship between the alterations in EGFR, HER2, TOP2A, chromosome 7, or chromosome 17 and the clinicopathologic characteristics of gastric carcinoma; this is in contrast to previous reports, and may be due to different sample numbers and different populations. Further studies will need to be performed using larger patient populations.

## Conclusion

This study documented alterations in protein expression and gene copy number for EGFR, HER2, and TOP2A in Chinese patients with gastric carcinoma. Alterations in HER2 and TOP2A occurred at a relatively low frequency. HER2 gene amplification correlated significantly with HER2 protein overexpression, whereas EGFR gene amplification and TOP2A gene amplification were not associated with corresponding increases in protein expression; the mechanism remains to be clarified. Chromosome 7 polysomy may contribute to an increase in EGFR gene copy number, and chromosome 17 polysomy may not be the main reason behind HER2 gene amplification. EGFR gene amplification did not correlate with HER2 gene amplification, whereas TOP2A gene amplification was associated significantly with HER2 gene amplification. Simultaneous detection of the status of the EGFR, HER2, and TOP2A genes may facilitate the development of effective therapeutic strategies to target gastric cancer, especially for determining those patients who may benefit most from a combination of therapies to target EGFR and HER2 together with a TOP2A inhibitor.

## Competing interests

The authors declare that they have no competing interests.

## Authors' contributions

LZ designed the experiment and drafted the manuscript, ZX participated in experiment design and performed FISH analysis, GJ performed immunohistochemistry staining, WS performed FISH analysis, WP collected patients information and participated in immunohistochemistry analysis, SX participated in statistically analysis. ZJ participated in specimen collection and FISH analysis, LT conceived of the study, and participated in its design and coordination.

## Pre-publication history

The pre-publication history for this paper can be accessed here:



## References

[B1] Corley DA, Buffler PA (2001). Oesophageal and gastric cardia adenocarcinoma: analysis of regional variation using the Cancer Incidence in FIVE Contiinents database. Int J Epidemiol.

[B2] Takehana T, Kunitomo K, Kono K, Kitahara F, Iizuka H, Matsumoto Y, Fujino MA, Ooi A (2002). Status of c-ERBB-2 in gastric adenocarcinoma: a comparative study of immunohistochemistry, fluorescence in situ hybridization and enzyme-linked immuno-sorbent assay. Int J Cancer.

[B3] García I, Vizoso F, Martín A, Sanz L, Abdel-Lah O, Raigoso P, García-Muñiz JL (2003). Clinical significance of the epidermal growth factor receptor and HER2 receptor in respectable gastric cancer. Ann Surg Oncol.

[B4] Costa MJ, Walls J (1996). Epidermal growth factor receptor and c-erbB-2 oncoprotein expression in female genital tract carcinosarcomas (malignant mixed mullerian tumors). Clinicopathologic study of 82 cases. Cancer.

[B5] Wright C, Angus B, Nicholson S, Sainsbury JR, Cairns J, Gullick WJ, Kelly P, Harris AL, Horne CH (1989). Expression of c-erbB-2 oncoprotein: a prognostic indicator in human breast cancer. Cancer Res.

[B6] Soh J, Toyooka S, Ichihara S, Fujiwara Y, Hotta K, Suehisa H, Kobayashi N, Ichimura K, Aoe K, Aoe M, Kiura K, Date H (2007). Impact of HER2 and EGFR gene status on gefitinib-treated patients with nonsmall-cell lung cancer. Int J Cancer.

[B7] Moulder SL, Yakes FM, Muthuswamy SK, Bianco R, Simpson JF, Arteaga CL (2001). Epidermal growth factor receptor (HER1) tyrosine kinase inhibithor ZD1839(Iressa) inhibits HER2/neu (erbB2)-overexpressing breast cancer cells in vitro and in vivo. Cancer Res.

[B8] Xia W, Mullin RJ, Keith BR, Liu LH, Ma H, Rusnak DW, Owens G, Alligood KJ, Spector NL (2002). Anti-tumor activity of GW572016: a dual tyrosine kinase inhibitor blocks EGF activation of EGFR/erbB2 and downstream Erk1/2 and AKT pathways. Oncogene.

[B9] Järvinen TAH, Liu ET (2006). Simultaneous amplification of HER-2(ERBB2) and Topoisomerase IIα (TOP2A) genes-molecular basis for combination chemotherapy in cancer. Current Cancer Drug Targets.

[B10] Mano MS, Rosa DD, Azambuja ED, Ismael GFV, Durdecq V (2007). The 17q12-q21 amplicon:Her2 and topoisomerase – IIα and their importance to the biology of solid tumors. Cancer Treatment Reviews.

[B11] Buckingham LE, Coon JS, Morrison LE, Jacobson KK, Jewell SS, Kaiser KA, Mauer AM, Muzzafar T, Polowy C, Basu S, Gale M, Villaflor VM, Bonomi P (2007). The prognostic value of chromosome 7 polysomy in non-small cell lung cancer patients treated with gefitinib. J Thorac Oncol.

[B12] Wang S, Saboorian MH, Frenkel EP, Haley BB, Siddiqui MT, Gokaslan S, Hynan L, Ashfaq R (2002). Aneusomy 17 in breast cancer: Its role in Her2/neu protein expression and implication for clinical assessment of Her-2/neu status. Mod Pathol.

[B13] Flemming ID, Cooper JS, Henson DE, Hutter RVP, Kennedy BJ, Murphy GP, O'Sullivan B, Sobin LH, Yarbro JW, editors (1997). AJCC cancer staging manual.

[B14] Fenoglio-Preiser C, Hamilton S, Aaltonen LA (2000). Tumours of the stomach. WHO classification of tumours.

[B15] Wolff AC, Hammond ME, Schwartz JN, Hagerty KL, Allred DC, Cote RJ, Dowsett M, Fitzgibbons PL, Hanna WM, Langer A, McShane LM, Paik S, Pegram MD, Perez EA, Press MF, Rhodes A, Sturgeon C, Taube SE, Tubbs R, Vance GH, Vijver M van de, Wheeler TM, Hayes DF, American Society of Clinical Oncology; College of American Pathologists American Society of Clinical Oncology/College of American Pathologists guideline recommendations for human epidermal growth factor receptor 2 testing in breast cancer. J Clin Oncol.

[B16] Cappuzzo F, Hirsch FR, Rossi E, Bartolini S, Ceresoli GL, Bemis L, Haney J, Witta S, Danenberg K, Domenichini I, Ludovini V, Magrini E, Gregorc V, Doglioni C, Sidoni A, Tonato M, Franklin WA, Crino L, Bunn PA, Varella-Garcia M (2005). Epidermal growth factor receptor gene and protein and gefitinib sensitivity in non-small-cell lung cancer. J Natl Cancer Inst.

[B17] Shin HJ, Shin DM, Tarco E, Sneige N (2003). Detection of Numerical Aberrations of Chromosomes 7 and 9 in Cytologic Specimens of Pleural Malignant mesothelioma. Cancer.

[B18] Kapranos N, Kounelis S, Karantasis H, Kouri E (2005). Numerical Aberrations of Chromosomes 1 and 7 by Fluorescent in Situ Hybridization and DNA Ploidy Analysis in Breast Cancer. The Breast Journal.

[B19] Kanta SY, Yamane T, Dobashi Y, Mitsui F, Kono K, Ooi A (2006). Topoisomerase IIα gene amplification in gastric carcinomas: correlation with the HER2 gene. An immunohistochemical, immunoblotting, and multicolor fluorescence in situ hybridization study. Human Pathology.

[B20] Park DI, Yun JW, Park JH, Oh SJ, Kim HJ, Cho YK, Sohn CI, Jeon WK, Kim BI, Yoo CH, Son BH, Cho EY, Chae SW, Kim EJ, Sohn JH, Ryu SH, Sepulveda AR (2006). HER-2/neu amplification is an independent prognostic factor in gastric cancer. Dig Dis Sci.

[B21] Varis A, Zaika A, Puolakkainen P, Nagy B, Madrigal I, Kollola A, Väyrynen A, Kärkkäinen P, Moskaluk C, El-Rifai W, Knuutila S (2004). Coamplified and overexpressed genes at ERBB2 locus in gastric cancer. Int J Cancer.

[B22] Galizia G, Lieto E, Orditura M, Castellano P, Mura AL, Imperatore V, Pinto M, Zamboli A, De Vita F, Ferraraccio F (2007). Epidermal Growth Factor Receptor (EGFR) Expression is Associated With a Worse Prognosis in Gastric Cancer Patients Undergoing Curative Surgery. World J Surg.

[B23] Jeona YK, Sungb SW, Chunga JH, Parke WS, Seoa JW, Kima CW, Chung DH (2006). Clinicopathologic features and prognostic implications of epidermal growth factor receptor (EGFR) gene copy number and protein expression in non-small cell lung cancer. Lung Cancer.

[B24] Suzuki S, Dobashi Y, Sakurai H, Nishikawa K, Hanawa M, Ooi A (2005). Protein overexpression and gene amplification of epidermal growth factor receptor in nonsmall cell lung carcinomas:an immunohistochemical and fluorescence in situ hybridization study. Cancer.

[B25] Onn A, Correa AM, Gilcrease M, Isobe T, Massarelli E, Bucana CD, O'Reilly MS, Hong WK, Fidler IJ, Putnam JB, Herbst RS (2004). Synchronous overexpression of epidermal growth factor receptor and HER2-*neu *protein is a predictor of poor outcome in patients with stage I non-small cell lung cancer. Clin Cancer Res.

[B26] Hirsch FR, Varella-Garcia M, Bunn PA, Di Maria MV, Veve R, Bremnes RM, Barón AE, Zeng C, Franklin WA (2003). Epidermal growth factor receptor in non-small-cell lung carcinomas: correlation between gene copy number and protein expression and impact on prognosis. J Clin Oncol.

[B27] Li TK, Liu LF (2001). Tumor cell death induced by topoisomerase-target drugs. Annu Rev Pharmacol Toxicol.

[B28] Ju BG, Lunyak VV, Perissi V, Garcia-Bassets I, Rose DW, Glass CK, Rosenfeld MG (2006). A topoisomereaseIIβ-mediated dsDNA break required for regulated transcription. Science.

[B29] Järvinen TAH, Tanner M, Rantanen V, Bärlund M, Borg A, Grenman S (2000). Amplification and deletion of topoisomerase IIαgene are common in ErbB-2 amplified breast cancer and alter the sensitivity to doxorubicin. Am J Pathol.

[B30] Varis A, Wolf M, Monni O, Vakkari ML, Kokkola A, Moskaluk C, Frierson H, Powell SM, Knuutila S, Kallioniemi A, El-Rifai W (2002). Targets of gene amplification and overexpression at 17q in gastric cancer. Cancer Res.

[B31] Simon R, Atefy R, Wagner U, Forster T, Fijan A, Bruderer J, Wilber K, Mihatsch MJ, Gasser T, Sauter G (2003). HER-2 and TOP2A coamplification in urinary bladder cancer. Int J Cancer.

[B32] Guerin E, Entz-Werle N, Eyer D, Pencreac'h E, Schneider A, Falkenrodt A, Uettwiller F, Babin A, Voegeli AC, Lessard M, Gaub MP, Lutz P, Oudet P (2003). Modification of topoisomerase genes copy number in newly diagnosed childhood acute lymphoblastic leukemia. Leukemia.

